# Skeletal Muscle Differentiation Evokes Endogenous XIAP to Restrict the Apoptotic Pathway

**DOI:** 10.1371/journal.pone.0005097

**Published:** 2009-03-31

**Authors:** Michelle I. Smith, Yolanda Y. Huang, Mohanish Deshmukh

**Affiliations:** Department of Cell and Developmental Biology and Neuroscience Center, University of North Carolina, Chapel Hill, North Carolina, United States of America; University of Helsinki, Finland

## Abstract

Myotube apoptosis occurs normally during muscle development and aging but it can lead to destruction of skeletal muscle in neuromuscular diseases. Therefore, understanding how myotube apoptosis is regulated is important for developing novel strategies for treatment of muscle loss. We investigated the regulation of apoptosis in skeletal muscle and report a striking increase in resistance to apoptosis following differentiation. We find mitotic C2C12 cells (myoblast-like cells) are sensitive to cytosolic cytochrome *c* microinjection. However, differentiated C2C12 cells (myotube-like cells) and primary myotubes are markedly resistant. This resistance is due to endogenous X-linked inhibitor of apoptotic protein (XIAP). Importantly, the selective difference in the ability of XIAP to block myotube but not myoblast apoptosis is not due to a change in XIAP but rather a decrease in Apaf-1 expression. This decrease in Apaf-1 links XIAP to caspase activation and death. Our findings suggest that in order for myotubes to die, they may degrade XIAP, functionally inactivate XIAP or upregulate Apaf-1. Importantly, we identify a role for endogenous Smac in overcoming XIAP to allow myotube death. However, in postmitotic cardiomyocytes, where XIAP also restricts apoptosis, endogenous Smac was not capable of overcoming XIAP to cause death. These results show that as skeletal muscle differentiate, they become resistant to apoptosis because of the ability of XIAP to regulate caspase activation. The increased restriction of apoptosis in myotubes is presumably important to ensure the long term survival of these postmitotic cells as they play a vital role in the physiology of organisms.

## Introduction

Skeletal muscle is a highly specialized tissue that is unique in its structure and development. Individual myotubes that comprise skeletal muscle are derived from mitotic myoblasts which under the right environmental cues begin to express myogenic markers, exit the cell cycle and fuse to form long multinucleated myotubes [1]. While the molecular details of this differentiation process are well understood, very little is known about whether fundamental biological processes such as apoptosis are altered during this process of differentiation. Understanding this phenomenon is important because, following the developmental period, muscle loss can have deleterious effects. For example, conditions such as muscular dystrophies, neurogenic muscular atrophy and mitochondrial myopathies result in skeletal muscle death involving apoptosis [2].

Apoptosis is a genetically regulated, evolutionarily conserved form of cell death. It is characterized by the activation of caspase proteases that cleave numerous substrates within the cell to cause the demise of the cell [3]. In the intrinsic pathway of apoptosis which can be activated by various stressors such as growth factor withdrawal, ER stress and DNA damage, signaling pathways converge upon the proapoptotic proteins Bax and Bak. This causes their activation and translocation to the mitochondria where they release cytochrome *c* from the intermembrane space. Once free in the cytosol, cytochrome *c* binds to the adapter protein Apaf-1. This binding induces a conformational change in Apaf-1 in such a way that Apaf-1 oligomerizes as well as binds to procaspase 9 to form the apoptosome complex. Once on the apoptosome, caspase 9 becomes active and cleaves procaspase 3 into its active form. Active caspase 3 is known as the executioner caspase because it cleaves various proteins ultimately leading to the death of the cell [4], [5].

Recent reports indicate that mitotic myoblasts utilize an alternative mechanism of activating caspases [6]. Activation of caspases 9 in these cells occurs independently of Apaf-1 but still requires release of endogenous Smac from the mitochondria [7], [8]. Smac is a mitochondrial intermembrane space protein which acts as an inhibitor of an antiapoptotic family of proteins known as the Inhibitor of Apoptotic Proteins (IAPs) [9], [10]. IAPs regulate apoptosis by binding to and inhibiting caspases [11]. Despite our knowledge of the structure and function of Smac, a critical role for endogenous Smac in regulating apoptosis has not been discovered in other primary cells. Importantly, what happens to this pathway upon differentiation of myoblasts into myotubes is unknown. Skeletal muscles are known to become more resistant to apoptosis upon differentiation [12]. However, as most of the studies examining skeletal muscle apoptosis have focused on whole tissue, the mechanism for this increased resistance has not been clearly identified at the cellular level.

In this study, we investigated how isolated myotubes regulate their caspase activation following differentiation. We report that myotubes exhibit an increase in their resistance to apoptosis relative to their mitotic precursor cells. While mitotic C2C12 cells (mC2C12) die with the introduction of cytochrome *c* into their cytosol, differentiated C2C12 (dC12C12) cells and primary myotubes do not. This increased resistance is due to endogenous XIAP. We show that endogenous XIAP is able to selectively block caspase activation in myotubes not because its levels are increased in myotubes but rather because the level of Apaf-1 is dramatically decreased. Importantly, our studies identify endogenous Smac as having a vital role in overcoming this XIAP inhibition in myotubes but not cardiomyocytes.

## Results

### Myotubes develop resistance to cytochrome *c*-induced apoptosis upon differentiation

**Figure 1:**
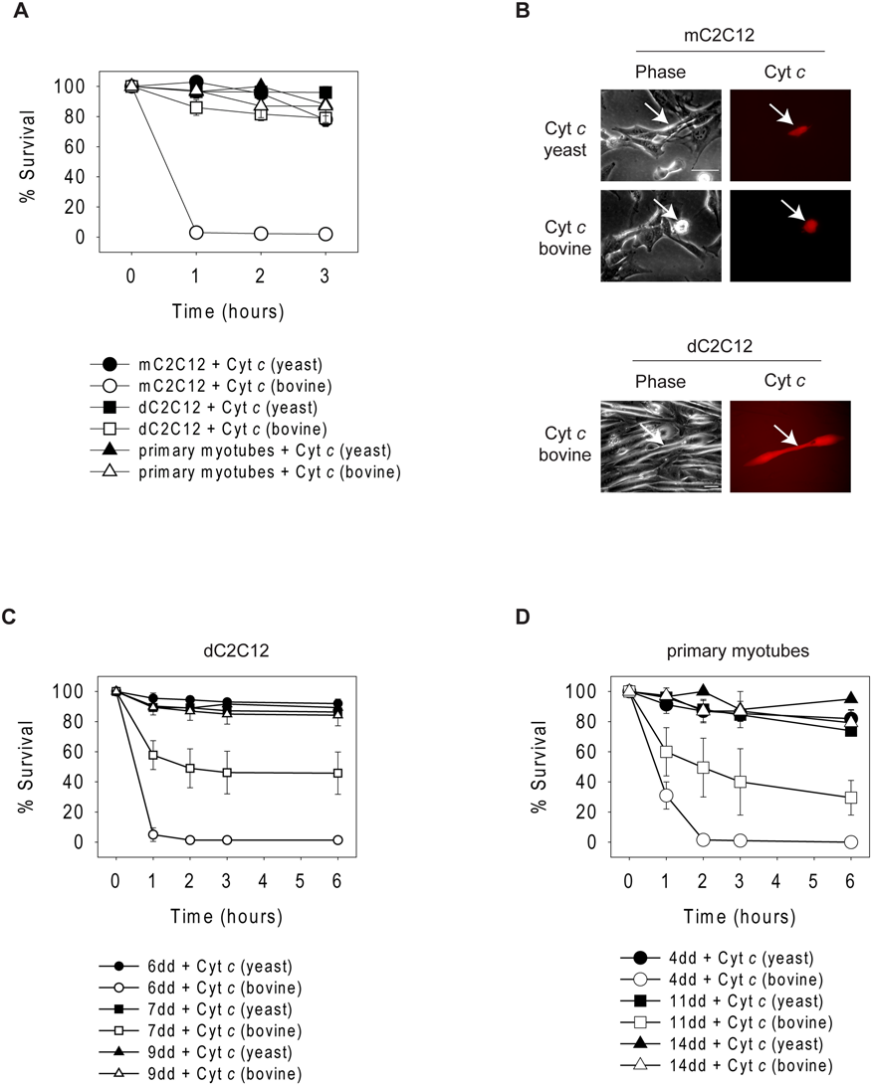
Myotubes develop gradual resistance to cytosolic cytochrome *c*-induced apoptosis. (A) mC2C12 cells, dC2C12 cells differentiated for 9 days and primary myotubes differentiated for 14 days, were injected with either yeast or bovine cytochrome *c* and rhodamine dextran. (B) Photographs of mC2C12 and dC2C12 cells one hour following injection with yeast or bovine cytochrome *c* and rhodamine dextran. Arrows point to injected cells. Scale bars represent 50 µm. (C) C2C12 cells differentiated for 6, 7 or 9 days (dd = days differentiated) were injected with either yeast or bovine cytochrome *c* and rhodamine dextran. (D) Primary myotubes differentiated for 4, 11 or 14 days were injected with either yeast or bovine cytochrome *c* and rhodamine dextran. Cell survival was assessed by morphology at the indicated times following injection. Data are the mean±SEM of n≥3 separate experiments per time point.

To determine whether cytochrome *c*-mediated caspase activation becomes more restricted with skeletal muscle differentiation, we microinjected cytochrome *c* in C2C12 cells and myotubes. Myoblast-like C2C12 (mC2C12) cells were very sensitive to bovine cytochrome *c* with almost a complete loss of injected cells within one hour (Fig. 1a, b). Yeast cytochrome *c* serves as an ideal control because unlike mammalian cytochrome *c*, it is not capable of binding to Apaf-1, and therefore, cannot activate the apoptosome [13]. As anticipated, mC2C12 cells injected with yeast cytochrome *c* did not die, indicating that microinjection alone was not killing these cells (Fig. 1a, b). In striking contrast to the mC2C12 cells, C2C12 cells that had been differentiated for nine days (dC2C12) did not undergo death when injected with bovine or yeast cytochrome *c* (Fig. 1a, b). To examine primary cells, we isolated murine myoblasts. While the small size of primary myoblasts made them technically difficult to microinject, we were able to differentiate them in culture for 14 days into myotubes and inject these myotubes. Just as seen with the dC2C12 cells, primary myotubes were markedly resistant to bovine cytochrome *c* (Fig. 1a). Together, this data indicated that while mC2C12 cells were sensitive to cytochrome *c* induced apoptosis, postmitotic dC2C12 cells and primary myotubes developed resistance.

During differentiation, myoblasts exit the cell cycle and fuse to form myotubes [1]. To determine at what point myotubes gained resistance to cytosolic cytochrome *c*, we injected dC2C12 cells and primary myotubes at different days of differentiation. Following six days of differentiation, C2C12 cells still remained sensitive to cytosolic cytochrome *c* injections, showing complete apoptosis one hour after injection. By seven days of differentiation they had developed some resistance and by nine days, almost all cells survived cytochrome *c* injection (Fig. 1c). Likewise, differentiating primary myotubes also showed a gradual resistance to cytochrome *c*. Four days into the differentiation process only 30 % survived one hour post cytochrome *c* injection. However, by 11 days this survival increased to about 60 % and almost complete protection was seen by 14 days (Fig. 1d). Injecting yeast cytochrome *c* did not induce death at any stage of differentiation. These data suggest that as myotubes differentiate they gradually develop resistance to cytochrome *c* and lose their ability to undergo apoptosis in response to cytochrome *c*.

### Myotube resistance to cytochrome *c* can be overcome with the exogenous addition of the IAP inhibitor Smac or genetic deletion of XIAP

**Figure 2:**
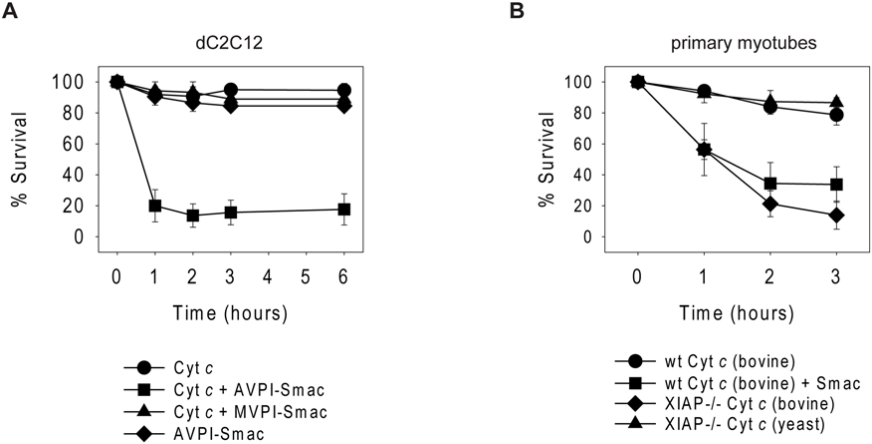
Resistance to cytosolic cytochrome *c* is mediated by endogenous XIAP. (A) dC2C12 cells were injected with rhodamine dextran and either bovine cytochrome *c*, wildtype AVPI-Smac, mutant MVPI-Smac or AVPI-Smac and cytochrome *c* together. (B) Primary wildtype (wt) myotubes were injected with rhodamine dextran and bovine cytochrome *c* or AVPI-Smac and bovine cytochrome *c*. XIAP-deficient (XIAP-/-) myotubes were injected with rhodamine dextran and either bovine or yeast cytochrome *c*. Cell survival was assessed by morphology at the indicated times following injection. Data are the mean±SEM of n≥3 separate experiments per time point.

Resistance to cytochrome *c* has been seen in primary neurons and cardiomyocytes where it has been linked to the function of endogenous XIAP [14]–[16]. To determine if IAPs also play a role in myotube resistance to cytochrome *c*, we injected both dC2C12 cells and primary myotubes with the IAP inhibitor Smac. When cytochrome *c* and Smac were coinjected into dC2C12 cells or primary myotubes, it resulted in rapid and complete death (Fig. 2). Control injections with cytochrome *c* or Smac alone did not induce significant death in these cells. To ensure that it was the IAP inhibiting function of Smac that was responsible for this action, we also injected cytochrome *c* into dC2C12 cells with a mutant form of Smac (MVPI-Smac) carrying a single point mutation that does not allow it to bind and inhibit IAPs [9]. In contrast to wildtype Smac (AVPI-Smac), coinjection of cytochrome *c* and mutant MVPI-Smac did not result in cell death (Fig. 2a).

These Smac injection experiments suggested that IAPs were in fact responsible for inhibiting cytochrome *c*-induced death in myotubes. Since XIAP has been shown to be the most effective IAP at inhibiting apoptosis [11], we tested whether endogenous XIAP was responsible for restricting myotube apoptosis. Myoblasts were isolated from wildtype and XIAP-deficient mice and differentiated in culture for 14 days prior to injection with cytochrome *c*. In contrast to wildtype myotubes, XIAP-deficient myotubes were strikingly sensitive to cytochrome *c* and underwent apoptosis by two hours following injection (Fig. 2b). XIAP-deficient myotubes injected with yeast cytochrome *c*, as a control, did not die. Taken together, these data suggest that myotubes are unable to undergo apoptosis in response to cytochrome *c* due to the strict control of caspase activation by endogenous XIAP.

### Endogenous XIAP effectively restricts cytochrome *c*-induced death in myotubes due to reduced Apaf-1 levels

**Figure 3:**
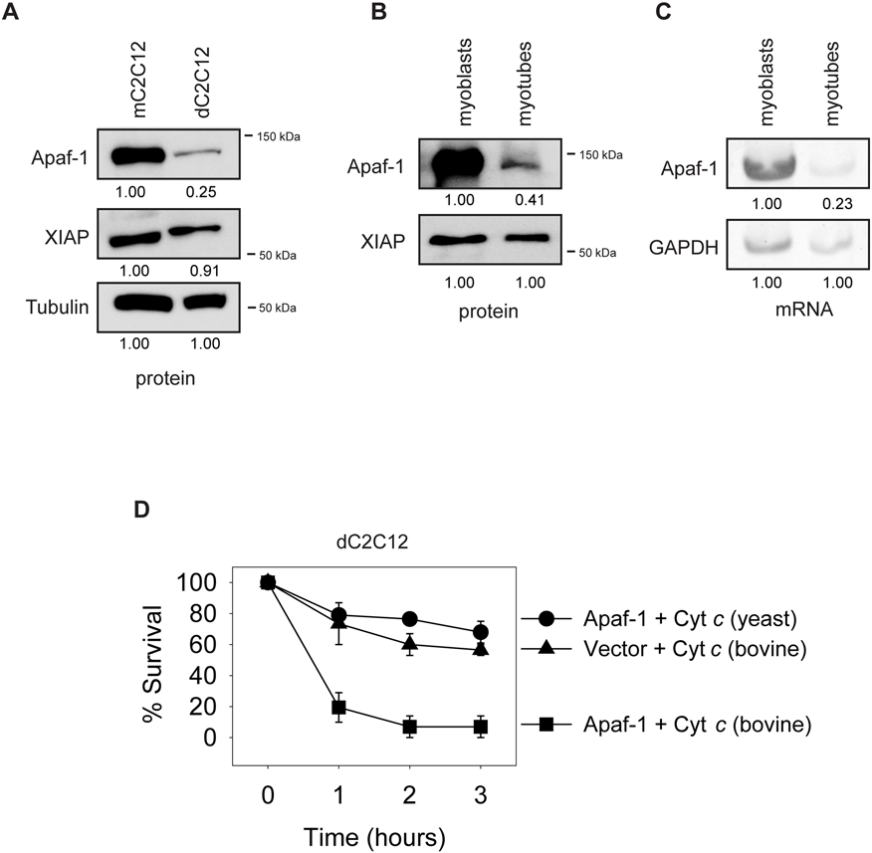
Apaf-1 levels are decreased in myotubes. Restoring Apaf-1 is sufficient to allow cytochrome *c*-mediated death. (A) Levels of the indicated apoptotic proteins were examined by Western blot of whole cell lysate from mC2C12 and dC2C12 cells. Tubulin serves as a loading control. Densitometry of protein levels are normalized to loading control protein levels of the representative Western blot. (B) Levels of the indicated apoptotic proteins were examined by Western blot of whole cell lysate from primary myoblasts and primary myotubes. Densitometry of protein levels are normalized to loading control protein levels of the representative Western blot. (C) RT-PCR was carried out with primers for the indicated mRNA using RNA from primary myoblasts and myotubes. GAPDH serves as a control. Densitometry of Apaf-1 mRNA levels are normalized to GAPDH levels of the representative gel. (D) dC2C12 cells were injected with plasmids for either Apaf-1 (Apaf) or empty vector as well as GFP. 24 h following injection, GPF positive cells were injected with rhodamine dextran and either yeast or bovine cytochrome *c*. Cell survival was assessed by morphology at the indicated times following cytochrome *c* injection. Data are the mean±SEM of n≥3 separate experiments per time point.

XIAP is a ubiquitously expressed protein [17]. To determine why postmitotic myotubes have selectively developed this XIAP brake in apoptosis, we looked at the level of XIAP in these mitotic and postmitotic cells. We found XIAP levels to be the same in mC2C12 versus dC2C12 and primary myoblasts versus primary myotubes (Fig. 3a, b). However, we found that Apaf-1 levels were decreased in both dC2C12 cells and primary myotubes relative to their mitotic precursors (Fig. 3a, b). Examination of the mRNA levels also showed a decrease in Apaf-1 in primary myotubes relative to myoblasts (Fig. 3c). These results lead us to examine whether Apaf-1 was limiting for caspase activation in myotubes. To test this, we injected plasmids for Apaf-1 and GFP in dC2C12 cells. Twenty four hours following injections, GFP expressing cells were injected with cytosolic cytochrome *c*. Expression of Apaf-1 alone in dC2C12 cells did not induce death and the cells remained resistant to control injection of yeast cytochrome *c*. In contrast, injection of bovine cytochrome *c* was able to induce death in the Apaf-1 overexpressing dC2C12 cells. Cells injected with vector and GFP showed significantly less death with bovine cytochrome *c* (Fig. 3d). Thus, expressing Apaf-1 was sufficient to allow cytochrome *c*-mediated death in myotubes. Together, these results suggest that the decreased levels of Apaf-1 in myotubes results in limited caspase activation, thus allowing endogenous XIAP to effectively protect against cytochrome *c*-mediated death. Consistent with this model, our results show that increasing Apaf-1 levels overcame this XIAP inhibition and rendered the myotubes sensitive to cytochrome *c* injections.

### Endogenous Smac can overcome XIAP inhibition in myotubes but not in cardiomyocytes

Despite this increase in resistance to apoptosis, myotubes undergo apoptosis during development [12] and in response to pathological stimuli [2]. Our experiments suggest that in order for myotubes to undergo apoptosis, they would not only have to release cytochrome *c* but also overcome the function of XIAP in order to become competent to die. This could occur by decreasing XIAP levels (Fig. 2b), upregulating Apaf-1 (Fig. 3d) or by functional inactivation of XIAP (Fig. 2). There are several known potential IAP inhibitors in cells, two of which, HtrA2 and Smac, are localized to the mitochondria. As structural studies have cast doubts on the IAP inhibitory activity of HtrA2 [18], we focused on Smac. Our data show that excess exogenous Smac is able to permit cytochrome *c*-mediated apoptosis in myotubes (Fig. 2), but whether the release of *endogenous* Smac is capable of doing so was unknown.

**Figure 4:**
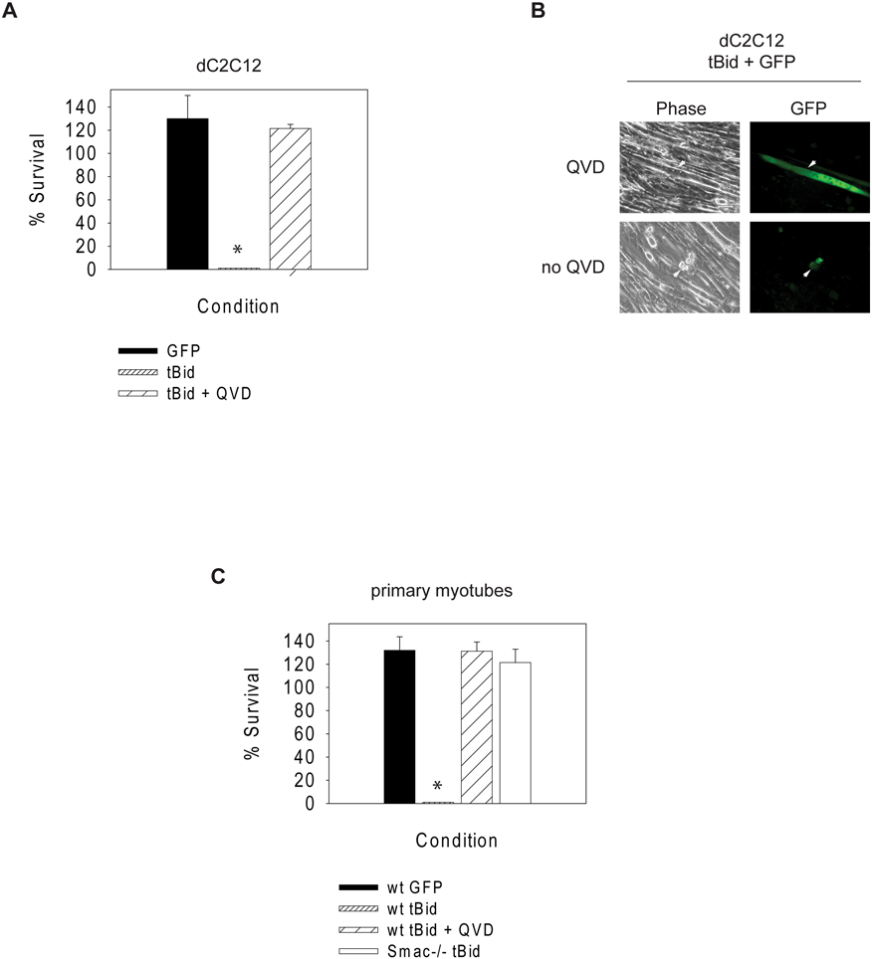
tBid causes caspase inhibitable death in myotubes that requires endogenous Smac. (A) dC2C12 were injected with plasmids for tBid or empty vector as well as GFP in the presence or absence of Q-VD-OPH (QVD). GFP expressing cells were counted 8 h and 24 h following injection. Percent survival was expressed as the percent of cells at 8 h that remained alive at 24 h following injection. Cell survival was assessed by morphology. Asterisk indicates that the actual survival was 0 %. (B) Photographs of dC2C12 cells eight hours following injection with tBid and GFP in the presence or absence of Q-VD-OPH (QVD). Arrows point to injected cells. Scale bars represent 50 µm. (C) Wild type (wt) and Smac-deficient (Smac-/-) primary myotubes were injected as described and assessment of survival was the same as in (A). Asterisk indicates that the actual survival was 0 %. Data are the mean±SEM of n≥3 separate experiments per time point.

To examine the importance of endogenous Smac in inhibiting XIAP and permitting apoptosis, we took advantage of the proapoptotic Bcl-2 family member tBid in order to release endogenous cytochrome *c*, Smac and other factors from the mitochondria (Fig. S1)[19]. Plasmids for tBid and GFP were injected into dC2C12 cells and primary myotubes and survival was assessed 24 hours later. Unlike cytochrome *c* injection, tBid expression induced potent death in these cells. This death was apoptotic as it was blocked with the pancaspase inhibitor Q-VD-OPH (Fig. 4a, b). These data suggest that tBid is able to release cytochrome *c* and presumably other mitochondrial factor(s) to permit a caspase-mediated apoptotic death in myotubes.

To directly determine if endogenous Smac was responsible for overcoming the function of XIAP in this tBid-mediated death, we isolated myoblasts from Smac-deficient mice. Once differentiated, we injected tBid and GFP into these myotubes. Unlike wildtype myotubes, Smac-deficient myotubes were completely resistant to tBid induced death (Fig. 4c). These results identify an important role for endogenous Smac in myotubes and imply that if adequate quantities of Smac were released from the mitochondria, this would be sufficient to overcome the XIAP brake and allow cytochrome *c* to induce myotube apoptosis.

**Figure 5:**
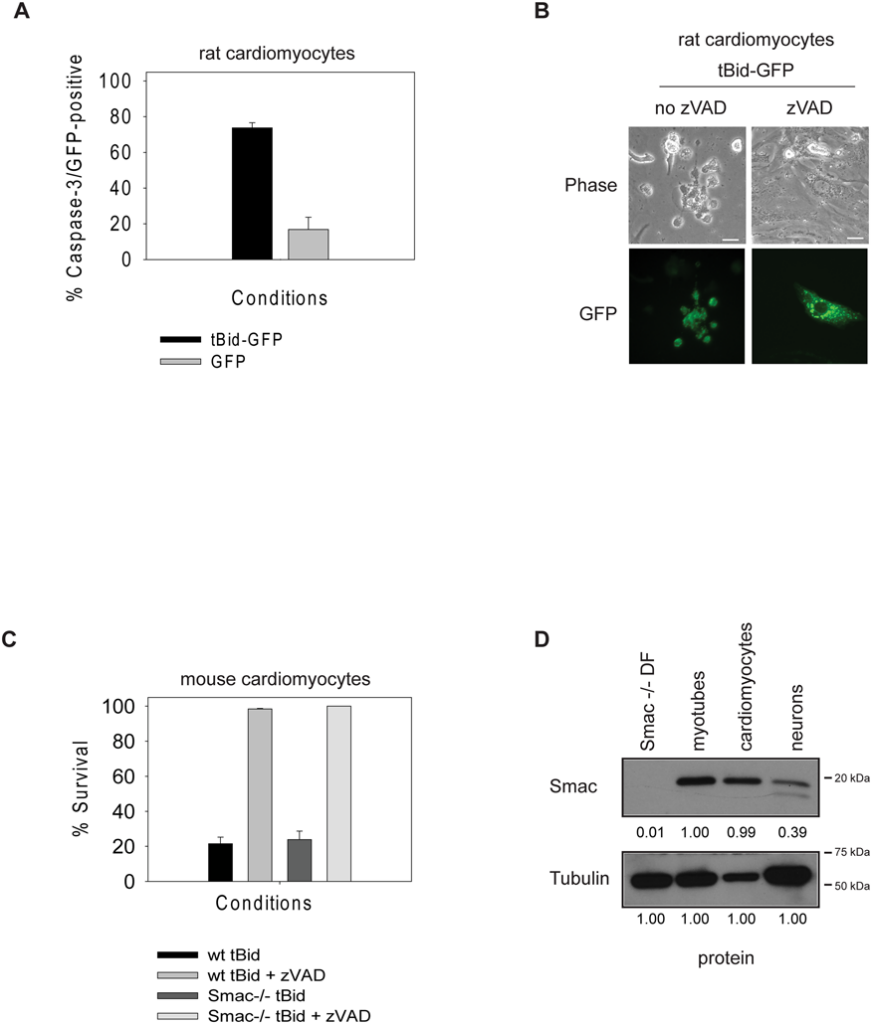
tBid induced apoptosis in cardiomyocytes does not require endogenous Smac. (A) Rat cardiomyocytes were transfected with tBid-GFP or GFP alone. The percentage of transfected cells expressing active caspase 3 was determined 6 hours after transfection by immunohistochemistry. (B) Photographs of rat cardiomyocytes 6 hours after transfection with tBid-GFP in the presence or absence of the caspase inhibitor z-VAD-fmk (zVAD). (C) Wild type (wt) or Smac-deficient (Smac-/-) cardiomyocytes were infected with a tBid-GFP adenovirus in the presence or absence of z-VAD-fmk (zVAD). Cell survival was determined by morphology over a 12 hour period using time-lapse microscopy. (D) Protein levels of Smac were examined by Western blot of whole cell lysates from Smac-deficient dermal fibroblasts (Smac-/- DF), myotubes, cardiomyocytes and sympathetic neurons (neurons). Tubulin serves as a loading control. Densitometry of Smac protein levels are normalized to tubulin levels of the representative blot.

We have previously reported that cardiomyocytes, like neurons and myotubes, utilize the XIAP brake to inhibit cytochrome *c*-induced apoptosis [16]. To determine if endogenous Smac is able to inhibit XIAP and permit apoptosis in cardiomyocytes, isolated neonatal rat cardiomyocytes were transfected with the tBid-GFP plasmid and GFP or GFP alone. Six hours after transfection the number of cells expressing active caspase 3 was determined by immunohistochemistry. While 70 % of tBid transfected cardiomyocytes showed active caspase 3 staining, this was seen in only 15 % of the GFP alone transfected cardiomyocytes (Fig. 5a). Photographs in Fig. 5b show that tBid transfected cardiomyocytes become rounded and died, whereas those in the presence of the pan caspase inhibitor z-VAD-fmk survived. Together, these data suggest that the release of cytochrome *c* and other mitochondrial proteins by tBid, was sufficient to cause caspase activation and apoptotic death in cardiomyocytes.

Next we determined if endogenous Smac was responsible for allowing tBid to induce death in cardiomyocytes, as seen in myotubes. Wildtype and Smac-deficient neonatal mouse cardiomyocytes were infected with a tBid-GFP adenovirus. Infected cells were examined by time-lapse microscopy over a 12 hour period. As expected, tBid-GFP infected wildtype cardiomyocytes showed only 20 % survival, and this death was blocked with the caspase inhibitor z-VAD-fmk. Surprisingly, and in contrast to mytotubes, Smac-deficient cardiomyocytes also showed only 23 % survival when infected with tBid-GFP adenovirus. The death of the Smac-deficient cardiomyocytes was apoptotic as it was completely blocked by the addition of z-VAD-fmk (Fig. 5c). Western analysis indicated that myotubes and cardiomyocytes expressed similar levels of endogenous Smac despite the fact that myotubes could utilize Smac to overcome XIAP whereas cardiomyocytes could not (Fig. 5d). Levels of Smac were lower in sympathetic neurons (Fig. 5d) which, like cardiomyocytes, do not appear to require endogenous Smac to overcome XIAP [14], [20].

Together, these data suggest that unlike myotubes, endogenous Smac was not required for mitochondria-mediated death in cardiomyocytes. In addition it suggests that there is likely to be an additional mitochondrial factor in cardiomyocytes that is released to allow XIAP to be overcome.

## Discussion

Together, the data presented in this study identify that postmitotic myotubes have increased suppression of their apoptotic pathway relative to their mitotic precursors. Differentiated C2C12 cells and primary myotubes were both resistant to cytosolic cytochrome *c* due to the activity of endogenous XIAP (Fig. 1, 2). However, myotubes inhibited caspase activation not by *increasing* XIAP levels but rather by *decreasing* Apaf-1 (Fig. 3a, b, c). Importantly, overexpressing Apaf-1 alone in dC2C12 cells, was sufficient to allow cytosolic cytochrome *c* injection to kill dC2C12 cells (Fig. 3d). Therefore, an Apaf-1 reduction is sufficient to set up this differential resistance between mitotic precursors and myotubes. It is noteworthy to mention that a previous study found that human skeletal muscle cytosol completely lacks Apaf-1 and therefore is refractory to cytochrome *c*-mediated caspase activation [21]. This difference could be attributed to the different model systems or the age of the skeletal muscle used, suggesting that skeletal muscle continue to reduce Apaf-1 expression with age.

Based on these data, we propose that following cytochrome *c* release from the mitochondria, the low levels of Apaf-1 present in myotubes result in reduced apoptosome formation and caspase activation. As a consequence, endogenous XIAP is sufficient to effectively block this reduced level of caspase activation. However, in mC2C12 cells, high levels of Apaf-1 result in robust apoptosome formation, and thus, increased caspase activation that cannot be successfully inhibited by endogenous XIAP. As a consequence, these mitotic cells readily undergo apoptosis with cytosolic cytochrome *c* while myotubes do not.

This increased resistance to apoptosis employed by myotubes is strikingly similar to that found in other postmitotic cells, specifically neurons and cardiomyocytes [14]–[16]. These findings suggest that regardless of the function or phenotype of the cell, postmitotic cells share the same features in restricting their ability to undergo apoptosis. By requiring not only cytochrome *c* release but also inhibition of XIAP function in order to activate caspases, endogenous XIAP presumably serves as a safety brake to death. For example, if the mitochondria accidentally release cytochrome *c*, the presence of XIAP would block caspase activation and therefore prevent these cells from undergoing unwanted apoptosis. Arguably, increased resistance to caspase activation would be critical to these postmitotic cells because of their inability to replicate and their limited regenerative potential. These postmitotic cells also serve vital functions that require their presence for the lifetime of the organism. On the other hand, not having this resistance to apoptosis in mitotic cells is beneficial to the organism as mitotic cells can give rise to cancer. Indeed, the mechanisms by which the apoptotic pathway is inactivated in many cancers are similar to those seen in postmitotic cells. For example, several melanomas restrict their Apaf-1 expression at the transcriptional level in order to block apoptosis [22]–[24]. In addition, many chemoresistant cancers have been found to utilize XIAP to effectively block caspase activation [25].

The XIAP-deficient mice lack an overt developmental phenotype [26]. This is consistent with our finding that myotube apoptosis is dependent on the removal of the endogenous XIAP brake as well as the release of cytochrome *c*. Neither event alone is capable of activating caspases. However, the XIAP-deficient myotubes are predicted to be more vulnerable if exposed to toxic stimuli or injury that causes mitochondrial damage and cytochrome *c* release, because they lack the postcytochrome *c* brake.

Despite the ability of myotubes to restrict apoptosis, there are certainly circumstances in which myotubes activate caspases and die. Caspase-mediated death has been observed during development [12]. The role of apoptosis in pathological conditions is more controversial. For example, several studies examining human spinal muscular atrophy found that muscle fibers experience apoptotic DNA fragmentation and the upregulation of apoptosis associated factors [27]–[29]. On the other hand, a study utilizing time-lapse microscopy to examine denervated and unloaded muscle did not reveal any nuclei loss in muscle fibers, suggesting apoptosis is not responsible for the loss of muscle mass experienced during muscle atrophy [30]. Caspase-mediated death is thought to be involved to some extent in additional pathological conditions including muscular dystrophies and mitochondrial myopathies [2].

Based on our findings, death stimuli that activate caspases in myotubes would not only have to cause the release of cytochrome *c* but also inhibit XIAP. Here we identified three ways in which this could occur. First, as XIAP-deficient myotubes are completely sensitive to cytosolic cytochrome *c* (Fig. 2b), a stimulus which is able to degrade or cleave XIAP and release cytochrome *c* would be able to sensitize myotubes to apoptosis. In fact, selective XIAP degradation has been seen in neurons undergoing apoptosis in response to nerve growth factor withdrawal [14]. Second, dC2C12 cells overexpressing Apaf-1 became sensitive to cytochrome *c*-mediated death (Fig. 3d). Therefore, a death stimulus which increases Apaf-1 expression would also be able to overcome XIAP inhibition. Supporting this idea, it has been shown that skeletal muscle undergoing caspase activation due to metabolic deficiencies with ATP depletion and oxidative stress show an increase in Apaf-1 levels [31]. Third, XIAP could also be functionally inhibited in response to a death stimulus. This could occur through several different mechanisms including an inactivating posttranslational modification of XIAP or via an endogenous inhibitor of XIAP. Here we show that releasing endogenous Smac from the mitochondria is indeed sufficient to overcome XIAP and allow cytochrome *c* to activate caspases in myotubes (Fig. 4c).

While the role of Smac as an IAP inhibitor has been extensively characterized *in vitro* [9], [10], the role of endogenous Smac has remained elusive since most cells do not need to inhibit XIAP to die [26], [32]. While neurons and cardiomyocytes engage the XIAP brake just like myotubes, one striking contrast between these postmitotic cells is that neurons and cardiomyocytes do not appear to utilize endogenous Smac to overcome XIAP (Fig. 5) [14], [20]. The levels of endogenous Smac are much lower in sympathetic neurons compared to myotubes (Fig. 5d). Therefore, it is possible that neurons do not contain enough Smac protein to fully inhibit XIAP. However, cardiomyocytes and myotubes have comparable levels of Smac (Fig. 5d), suggesting that the reason endogenous Smac is not effective in cardiomyocytes is not due to a lack of protein altogether. Interestingly, other than myotubes, the only cell type in which a role for endogenous Smac has been identified is their precursor, myoblasts [8]. This raises the intriguing possibility that endogenous Smac may play a vital role in this particular myogenic cell lineage but not in other cell types.

It is intriguing that Smac-deficient cardiomyocytes are still able to undergo tBid-induced death. This could be due to the release of an additional mitochondrial factor in cardiomyocytes. Known mitochondrial factors include AIF and HtrA2. AIF can be released from the mitochondrial and cause death but AIF-induced death is caspase independent [33] and therefore would not be blocked by the addition of z-VAD-fmk. Structural data have cast doubt on the IAP inhibitor function of HtrA2 [18], making it an unlikely candidate as well. This raises the possibility that cardiomyocytes contain an additional potentially novel mitochondrial factor that can overcome XIAP when released.

The potential significance of the XIAP brake in myotubes can be seen in mitochondrial encephalomyopathies, a group of heterogeneous disorders due to mutations in either mitochondrial DNA or nuclear genes. These mutations lead to mitochondrial abnormalities which ultimately result in a decrease in ATP synthesis and increased oxidative stress. Due to the heterogeneous nature of these deficiencies even within the same patient, some fibers appear healthy, some appear to suspend apoptosis and others seem to die with active caspase 3 immunoreactivity [31], [34], [35]. Many fibers from mitochondrial encephaolmypothaties show clear Bax upregulation and cytochrome *c* release. However, the number of fibers that show TUNEL staining is much lower [31], suggesting that in these fibers cytochrome *c* is released but cannot undergo caspase-mediated death. Our data would predict that in these fibers, it is XIAP that is able to prevent apoptosis despite the mitochondrial release of cytochrome *c*. A potential role of endogenous Smac can be seen *in vivo* as well, in patients with neurogenic muscular atrophy. In this condition, where patients experience apoptotic muscle fiber loss, there is an upregulation of multiple IAPs, including XIAP, but also Smac which appears to become released into the sarcoplasm [36]. Our findings would suggest that this release of Smac would be able to overcome XIAP and allow the affected muscle fibers to die.

## Materials and Methods

### Reagents

All reagents were purchased from Sigma-Aldrich or Fisher scientific, unless otherwise stated. Q-VD-OPH was purchased from MP Biomedicals. Protease inhibitor cocktail was purchased from Roche. XIAP-deficient mice were obtained from Dr. Craig B. Thompson (University of Pennsylvania) and Smac-deficient mice were obtained from Dr. Tak W. Mak (University of Toronto). Our procedure for genotyping these mice has been described previously [14], [20]. All the work involving animals was conducted using protocols approved by the Institutional Animal Care and Use Committee at UNC Chapel Hill and carried out under the regulations provided by the institutional body.

### C2C12 cell line and primary myoblast cultures

C2C12 cell line was maintained in DMEM containing 20 % FBS, 200 U/ml penicillin and 200 µg/ml streptomycin. When C2C12 cultures reached 70–90 % confluency they were differentiated by changing the media to differentiation medium consisting of DMEM supplemented with 2 % horse serum, 200 U/ml penicillin and 200 µg/ml streptomycin.

Satellite cell derived primary myoblasts were isolated from lower hindlimb muscle from mice ranging in age from two to four weeks old as described previously [37]. The primary cultures were maintained on collagen-coated dishes in Ham's F10 supplemented with 20 % FBS, 2.5 ng/ml bFGF, 200 U/ml penicillin, 200 µg/ml streptomycin, and 0.002 % Fungizone. The medium was changed every other day and cultures were differentiated with the addition of differentiation media when they reached 60–70 % confluency. All experiments were performed using primary cultures that had undergone between four and twelve passages. Experiments were performed on dC2C12 cells following 9 days of differentiation and on primary myotubes following 14 days of differentiation unless otherwise indicated.

### Primary cardiomyocyte cultures

Primary cardiomyocyte cultures were isolated from postnatal day 0–1 mice or rats using the Worthington neonatal cardiomyocyte isolation system (Worthington Biochemical Corp.) following the manufacturer's instructions. A 2 hour preplating step was included to reduce the number of fibroblasts in the cultures. Cells were then plated on laminin-coated MatTek 35 mm glass bottom dishes for time-lapse imaging or laminin-coated 35 mm dishes for transfection. Cells were grown in MEM with Earle's salt supplemented with 2 mM glutamine, 10 % horse serum, 5 % FBS, 100 U/ml penicillin, and 100 µg/ml streptomycin. Experiments were performed on rat cardiomyocytes 5 days after plating and mouse cardiomyocytes 2 days after plating. Rat cardiomyocytes were transfected with the indicated plasmid DNA using Lipofectamine 2000 (Invitrogen) following the manufacturer's instructions.

### Primary Smac-/- dermal fibroblast cultures

Primary dermal fibroblasts were isolated from postnatal day 0 (P0) Smac-deficient mice. The dorsal skin was removed, minced and rinsed in ice cold PBS. Tissue was then treated with 1 mg/ml collagenase, followed by digestion with 2.5 mg/ml trypsin for 1 hour each at 37°C. Tissue was then passaged through a 25 gauge needle until a single cell suspension was obtained. Cells were plated in DMEM with 10 % FBS, 100 mg/ml penicillin, and 100 mg/ml streptomycin.

### Primary sympathetic neuron cultures

Primary sympathetic neurons were isolated as described previously [38]. Briefly, superior cervical ganglia were dissected from P0 mice and treated with 1 mg/ml collagenase, followed by 2.5 mg/ml trypsin for 30 minutes each at 37°C. Cells were then dissociated by passaging through a fire-polished pipet and plated on collagen-coated dishes in MEM with Earle's salts supplemented with 50 ng/mL NGF, 10 % FBS, 2 mM glutamine, 100 µg/mL penicillin, 100 µg/mL streptomycin, 20 µM flourodeoxyuridine, 20 µM uridine, and 3.3 µg/ml aphidicolin. Experiments were performed on sympathetic neurons 5 days after plating.

### tBid-GFP Adenovirus production and use

As mouse cardiomyocytes are difficult to transfect, we generated an adenovirus expressing tBid-GFP. The recombinant virus Ad-tBid-GFP generated is responsive to TetR regulation and can be amplified in 293TREx cells without cytotoxicity. The *Mlu*I-*Xba*I fragment of pcDNA4/TO (Invitrogen) containing the CMV promoter with TetO was ligated into the *Mlu*I-*Nhe*I-digested pShuttle2 (BD Bioscience) to create pShuttle2/TO. tBid-GFP was PCR amplified with *Dra*I-*Xba*I ends from a plasmid from Dr. Douglas R. Green (St. Jude Children's Research Hospital) and cloned into pShuttle2/TO. Subsequent steps in generating the recombinant virus were according to manufacturer's instructions of BD Bioscience Adeno-X Expression System. The purification procedures and titer determination have been described [39]. In brief, Ad-tBid-GFP virus was purified by CsCl density gradient centrifugation. Viral titer was determined by an indirect immunofluorescent assay specific for the viral 72-kDa E2 gene product and defined as focus forming units (ffu) per ml. Neonatal cardiomyocytes were treated with Ad-tBid-GFP with multiplicity of infection (MOI) of 200 ffu per cell in a total volume of 100 µl for 3 hours before flooding the dishes with additional media to begin live imaging.

### Microinjection

Cells were plated on 35 mm dishes and microinjected with needles pulled on a Flaming-Brown horizontal micropipette puller (Sutter Instruments) using a Narashigi micromanipulator mounted on a Leica inverted florescent microscope. Between 25 and 100 cells were injected in each experiment. The microinjection buffer contained 100 mM KCl and 10 mM KP_i_, pH 7.4. For injections involving plasmid DNA, cells were injected and allowed 24 hours to express plasmid DNA prior to experimentation. DNA microinjections contained 50 ng/µl enhanced GFP (Clontech) and 200 ng/µl of the indicated plasmid. Cytochrome *c* microinjectons contained 5 mg/ml rhodamine dextran to mark injected cells and 25 ug/ul cytochrome *c*. Where indicated, 1 mg/ml recombinant Smac protein was injected along with 25 ug/uL cytochrome *c*. Following injections, viable cells were identified as rhodamine positive and intact. Data shown are mean±SEM of three independent experiments.

### Quantitation of cell survival

Cell survival after any treatment was assessed by counting clearly identifiable cells with intact morphology, whereas dead cells atrophied and degenerated. Surviving cells in the culture were counted and expressed as a percent of the number of cells in the 0 hour condition. This method of assessing survival correlates well with other cell survival assays such as trypan blue exclusion and staining with calcein AM [14].

### Western blots

Western blots were performed as previously described [14]. Primary antibodies were as follows: anti-Apaf-1 (Alexis), anti-XIAP (MBL), anti-Smac (R&D Systems) anti-alpha tubulin (Sigma). Mouse/rabbit/goat/rat HRP conjugated secondary antibodies were purchased form Pierce Chemical Co. Western blots were developed using the ECL-Plus detection system (Amersham Biosciences). Densitometry was performed using ImageJ software (NIH) and normalized to loading control protein levels of the representative Western blot.

### Quantitative RT-PCR analysis

Our method of quantitative RT-PCR analysis is a modification of a previously published protocol [40], where we substituted the radioactivity-based detection method with a fluorescence-based detection technique. Briefly, RNA was isolated from cells with DNAeasy kit (Qiagen) using the manufacturer's protocol. Equal amounts of the RNA isolated at specific times after the specified treatment was converted into cDNA with SuperScript II Reverse Transcriptase (Invitrogen). One microliter of cDNA was the template in a PCR using the following primer pairs:

APAF-1: Forward 5′ GAG GCA CAA TGG ATG CAA AGG 3′; Reverse 5′ GGC TGC TCG TTG ATA TTG AGT GG 3′

GAPDH: Forward 5′ CCA TGG AGA AGG CTG GGG 3′; Reverse 5′ CAA AGT TGT CAT GGA TGA CC 3′

Preliminary experiments validated that the RT-PCR technique was linear with respect to the amount of input RNA used for RT and with respect to the amount of cDNA used for PCR in these experiments. No product was amplified when water was used as input for a PCR reaction. Results were repeated in at least three independent RNA preparations. Levels were quantified using SYBR Green I Nucleic Acid Gel Stain (Molecular Probes Inc., Eugene, OR) and scanning blots on a Typhoon scanner (Amersham Biosciences). Densitometry was performed using ImageJ software (NIH) and normalized to GAPDH levels of the representative gel.

### Image acquisition and processing

Images were acquired by a Hamamatsu ORCA-ER digital B/W CCD camera mounted on a Leica inverted fluorescence microscope (DMIRE 2). The image acquisition software was Metamorph version 5.0 (Universal Imaging Corporation). Images were scaled down and cropped in Adobe Photoshop to prepare the final figures.

### Live-imaging of cardiomyocytes

Cardiomyocytes were imaged by a Zeiss Pascal confocal microscope in a live incubation chamber. Eight random regions in the dish were selected to be repeatedly imaged every 6 minutes for up to 12 hours. The survival of GFP-expressing neonatal cardiomyocytes was assessed by cell morphology using the Zeiss LSM image browser.

### Immunohistochemical analysis

Immunohistochemical analysis was performed using the same method as previously described [41]. Primary antibodies were as follows: anti-cytochrome *c* (BD Biosciences) and anti-GFP (Upstate Biotechnology Inc.). Secondary antibodies used were: anti-mouse CY3-conjugated (Jackson Immunoresearch Laboratories Inc.) and anti-chicken Alexa488-conjugated (Molecular Probes Inc.).

## Supporting Information

Figure S1 tBid induces the release of cytochrome c from mitochondria in differentiated C2C12 (dC2C12) cells. dC2C12 cells were injected with plasmids for tBid or empty vector, as well as GFP, in the presence of the caspase inhibitor Q-VD-OPH (to prevent cell death). 24 h after the injections, cells were immunostained with an antibody to cytochrome c. Arrows point to the injected cells. Control GFP alone expressing cells show cytochrome c staining (upper panel) which is lost upon its release from the mitochondria in tBid expressing cells (lower panel).
(2.39 MB TIF)
