# Supplementary figures and images for: Skeletal Muscle Differentiation Evokes Endogenous XIAP to Restrict the Apoptotic Pathway
Source: PLoS One. 2009 Mar 31;4(3):e5097. doi: 10.1371/journal.pone.0005097 (PMC2658743; doi:10.1371/journal.pone.0005097)

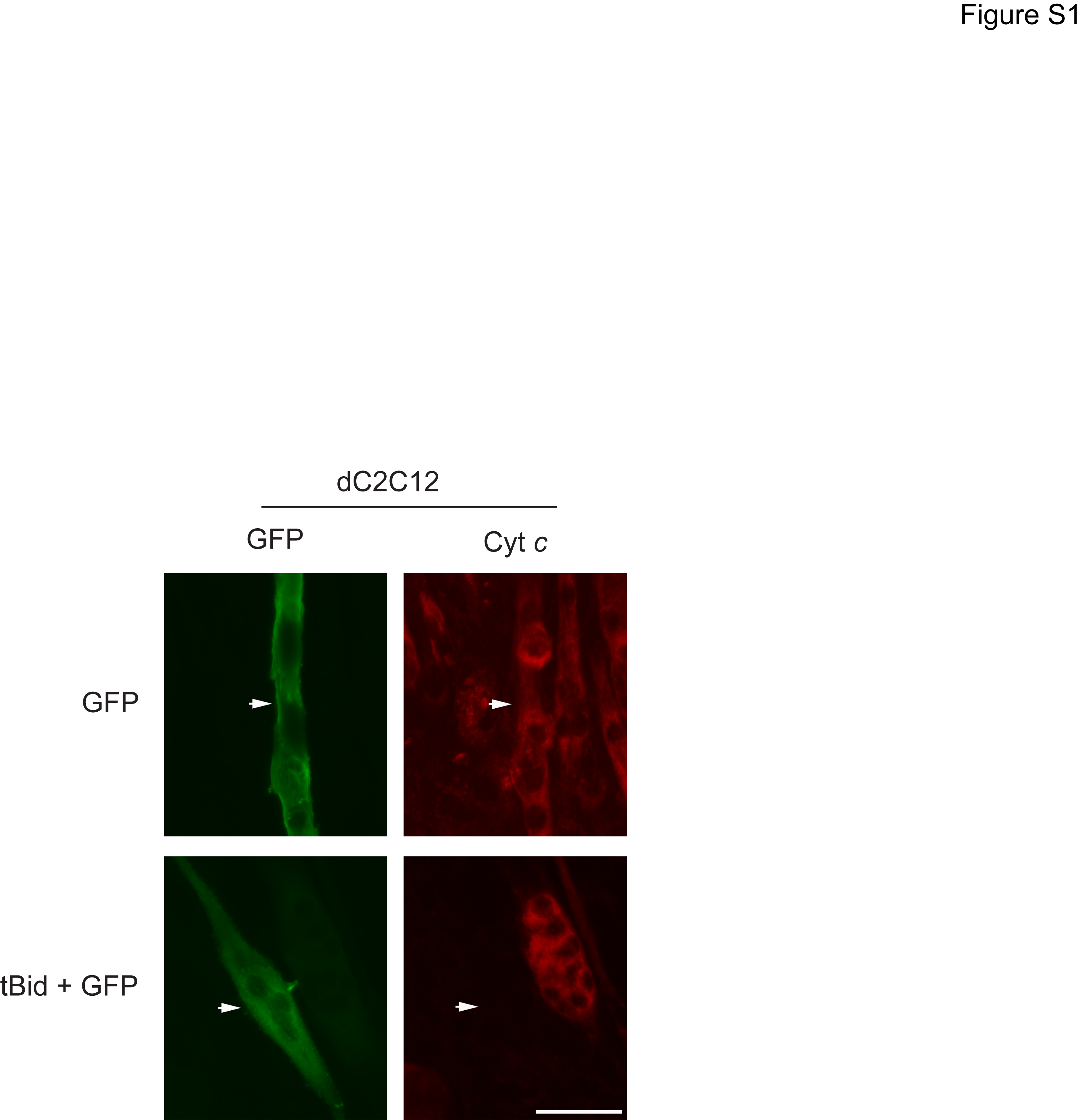

Supplement: Figure S1 — tBid induces the release of cytochrome c from mitochondria in differentiated C2C12 (dC2C12) cells. dC2C12 cells were injected with plasmids for tBid or empty vector, as well as GFP, in the presence of the caspase inhibitor Q-VD-OPH (to prevent cell death). 24 h after the injections, cells were immunostained with an antibody to cytochrome c. Arrows point to the injected cells. Control GFP alone expressing cells show cytochrome c staining (upper panel) which is lost upon its release from the mitochondria in tBid expressing cells (lower panel). (2.39 MB TIF) [file pone.0005097.s001.tif]
